# Supplementary material for: The First Whole Genome Sequence Discovery of the Devastating Fungus Arthrinium rasikravindrae
Source: J Fungi (Basel). 2022 Mar 2;8(3):255. doi: 10.3390/jof8030255 (PMC8954856; doi:10.3390/jof8030255)
Supplement: Supplementary file 1 [file jof-08-00255-s001.zip › jof-1594897-supplementary.pdf]

The First Whole Genome Sequence Discovery of the Devastating Fungus *Arthrinium rasikravindrae*

Supplementary data

Table of content

Supplement Fig/Table#	Tittle of figure	Page #
Supplementary Table S1	Statistical assembly summary for comparative fungal genomes…………………………………………	01
Supplementary Figure S1	NR annotation of species…………………………..	01
Supplementary Figure S2	TCBD functional classification…………………….	02
Supplementary Figure S3	Carbohydrate enzyme classification and annotation.	02
Supplementary Figure S4	Secondary metabolic gene Cluster…………………	03
Supplementary Figure S5	PHI phenotype classification………………………..	04

Supplementary Table S1. Statistical assembly summary for comparative fungal genomes.

Species	Naming	Accession number in Gene Bank	Total Length (MB)	GC (%)	N50	Scaffolds	Contigs
Arthrinium phaeospermum	AP-Z13	QYRS00000000.1	48.45	53.05	3,733,262	19	19
Arthrinium malaysianum	STlab-iicb	QUSE00000000.1	46.11	52.50	336,634	43	266
Fusarium proliferatum	ET1	FJOF00000000	45.21	48.50	3,311,891	32	17
Fusarium oxysporum	FO2	AAXH00000000.1	61.38	48.42	95,416	114	1362

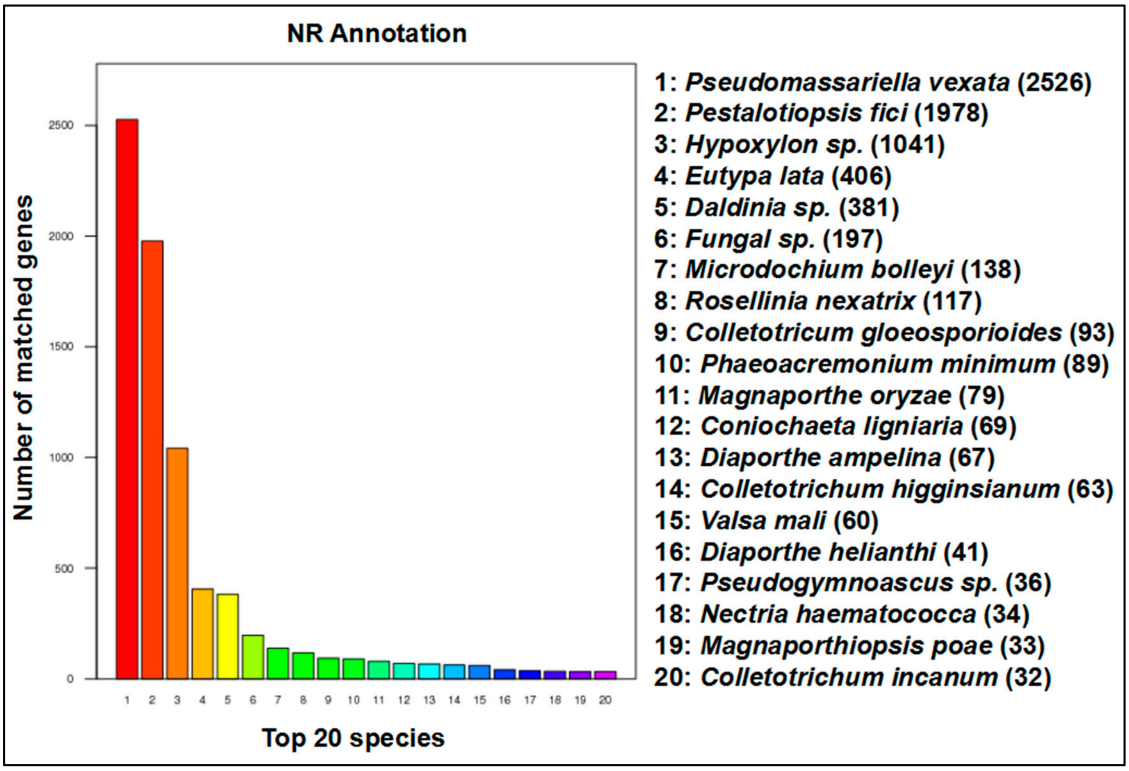

Supplementary Figure S1. NR annotation of species. In this figure, X-axis represents the top 20 species from 1-20, while the Y axis represents the number of matched genes between the species with a scale up to 2500. NR annotation is divided into 20 classes. Different colors depict top 20 species, their names, and values.

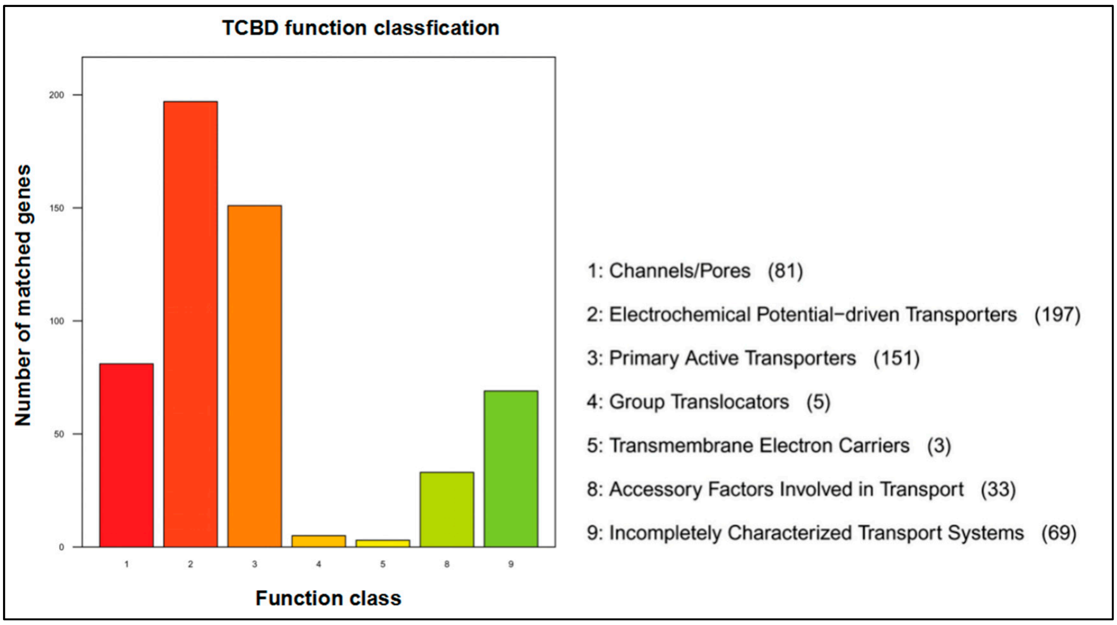

Supplementary Figure S2. TCBD functional classification. In this figure, X-axis represents the functional class & the Y-axis shows the number of matched genes, (a) TCBD functional classification 1-5, 8, and 9 class with different colors, functional class numbers, names, and values.

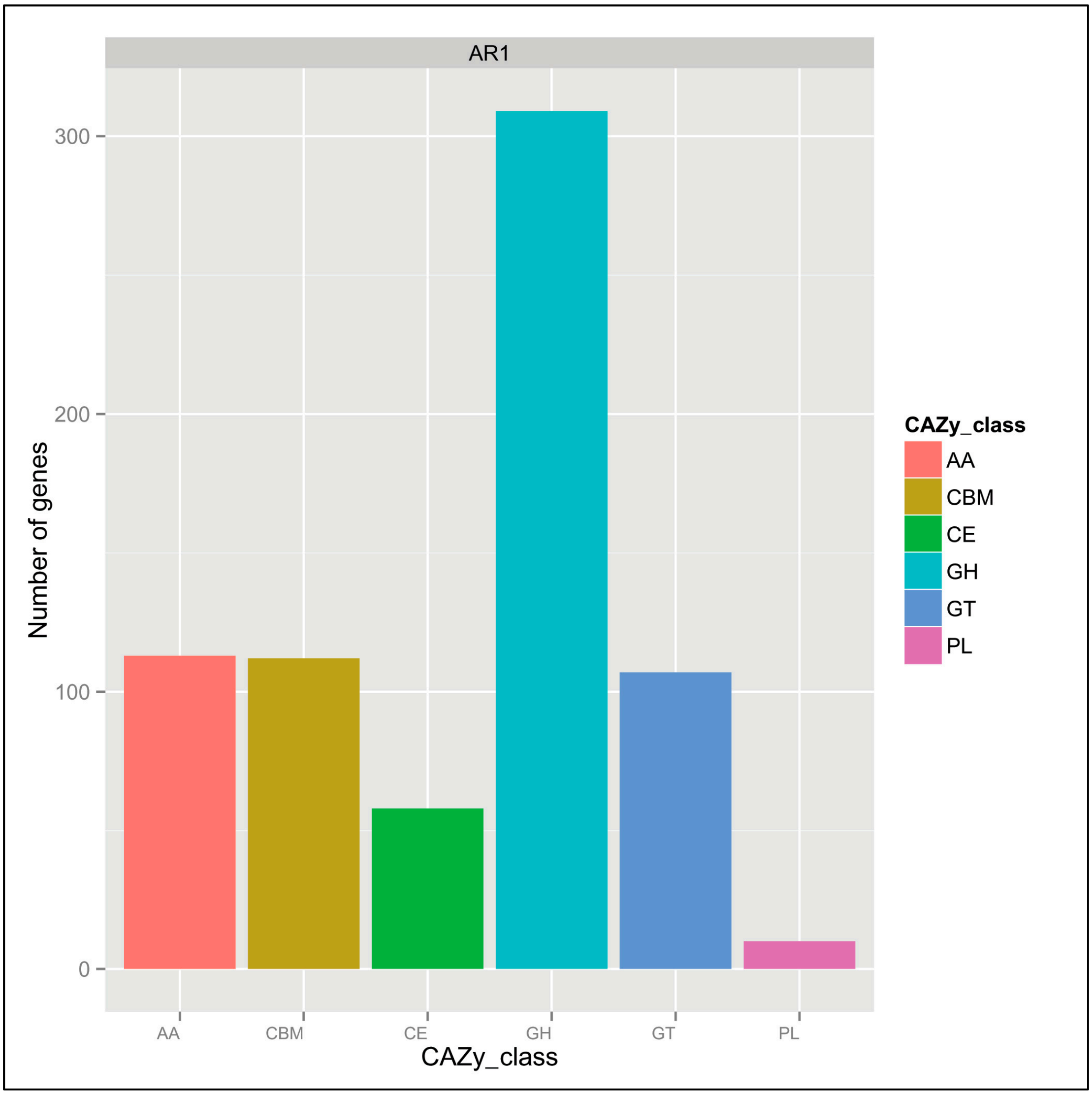

Supplementary Figure S3. Carbohydrate enzyme classification and annotation. In this figure X-axis shows the CAZy_class with their names as shown in different colors and Y-axis denotes the genes number of genes with a scale of 300, while it is divided into 6 classes (AA), (CBM), (CE), (GH), (GT) and (PL).

Note: AR1= *Arthrinium rasikravindrae* 1.

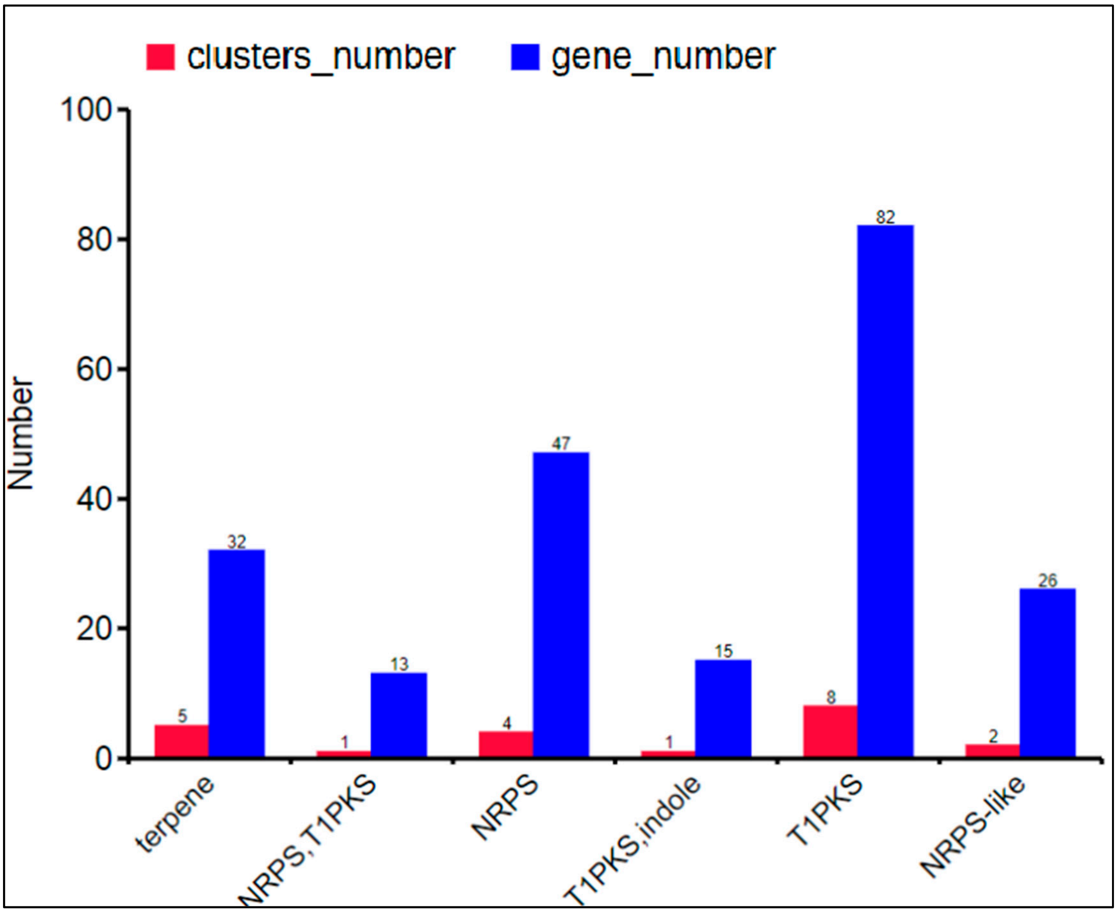

Supplementary Figure S4. Secondary metabolic gene Cluster. In this figure two different colors are shown, red color indicates the cluster number and blue color represents the gene number, while the X-axis denotes the type of cluster and their names, and the Y-axis shows gene numbers with a scale from 0-100.

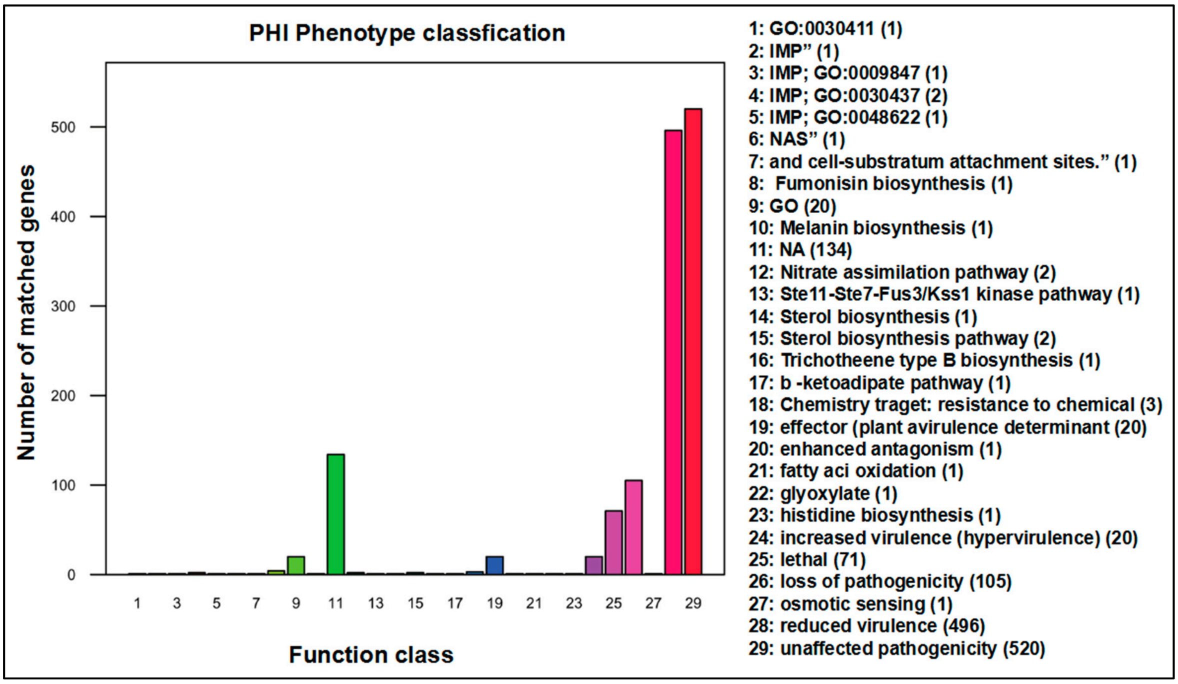

Supplementary Figure S5. PHI phenotype classification. In this figure, X-axis shows the function class with their class number and Y-axis shows the number of matched genes. PHI Phenotype classification shows 29 functional classes, functional class number and their names.
